# Formula feeding for late-preterm infants

**DOI:** 10.1186/1824-7288-40-S2-A37

**Published:** 2014-10-09

**Authors:** Luigi Corvaglia, Arianna Aceti

**Affiliations:** 1 Neonatology and Neonatal Intensive Care Unit S.Orsola-Malpighi Hospital, Department of Medical and Surgical Sciences (DIMEC), University of Bologna, Italy

## 

Preterm birth interrupts physiological foetal development, leading to various degrees of immaturity according to the gestational age at which the infant is born [1].

Since 2005, the imprecise definition of “near-term” infants has been replaced with “late-preterm”, which includes infants born between 34^0/7^ and 36^6/7^ weeks of gestation [2]. Late-preterm infants are at higher risk than term infants of developing medical complications that result in higher rates of mortality and morbidity [3], including thermal instability, respiratory problems, hypoglycaemia, jaundice, and feeding problems.

Breastfeeding is the first nutritional choice for all infants, especially for those born preterm. The establishment of successful breastfeeding in late-preterm infants is usually problematic, as late-preterm infants can be sleepier, have less muscular strength and more difficulty with latch, suck and swallow than term infants [4]. For this reason, health-care providers should implement specific strategies aimed at anticipate, identify promptly, and manage breastfeeding problems that the late-preterm infant and mother can experience.

However, when exclusive breastfeeding does not guarantee adequate nutrition, supplements might be advisable. Nutritional requirements of late-preterm infants are currently derived from speculations on foetal growth and requirements of preterm and term infants, while specific data on nutritional needs of this population are scarce. There is currently no consensus on whether late-preterm infants would benefit most of a high-protein diet, such as that proposed for “micropreterm” infants [5], or of a low-protein diet, such as that recommended for full-term infants. Some studies suggest that the provision of extra protein and energy could reduce weight loss and increase growth velocity [6], thus decreasing the risk for dehydration and hospital readmission. However, it is important to note that growth rate during late gestation decreases dramatically, and it is likely that protein and energy requirements for infants born during this period wouldn’t be as high as those of very preterm infants [7].

Current guidelines recommend the supplementation with essential nutrients also for late-preterm infants. Actually, it has been shown that supplementation with LC-PUFAs improves visual acuity and cognitive development in infants 30-37 weeks gestation [8].

The best nutritional approach to late-preterm infants still needs to be determined. Human milk’s benefits are undoubted; however, caregivers have to adequately support the establishment of successful breastfeeding and also identify those cases where some supplementation is needed. Further studies will have to clarify whether all late-preterm infants, or only a subgroup such as small-for-gestational-age infants, could benefit from formulas with high energy and protein content.
